# Olfactory and Gustatory Outcomes in COVID-19: A Prospective Evaluation in Nonhospitalized Subjects

**DOI:** 10.1177/0194599820939538

**Published:** 2020-06-30

**Authors:** Alberto Paderno, Davide Mattavelli, Vittorio Rampinelli, Alberto Grammatica, Elena Raffetti, Michele Tomasoni, Tommaso Gualtieri, Stefano Taboni, Silvia Zorzi, Francesca Del Bon, Davide Lombardi, Alberto Deganello, Luca Oscar Redaelli De Zinis, Alberto Schreiber

**Affiliations:** 1 Unit of Otorhinolaryngology–Head and Neck Surgery, ASST Spedali Civili di Brescia, Department of Medical and Surgical Specialties, Radiologic Sciences, and Public Health; University of Brescia, Brescia, Italy; 2 Epidemiology and Public Health Intervention Research group (EPHIR), Department of Global Public Health, Karolinska Institutet, Stockholm, Sweden

**Keywords:** COVID-19, SARS-CoV-2, olfactory dysfunction, gustatory dysfunction, smell, taste, recovery

## Abstract

**Objective:**

To prospectively assess the rate and timing of recovery of olfactory (OD) and gustatory (GD) dysfunction in patients affected by COVID-19.

**Study Design:**

Cohort study.

**Setting:**

Population-based evaluation in a COVID-19 high-prevalence region.

**Subjects and Methods:**

We analyzed the clinical course of OD and GD in a cohort of home-quarantined SARS-CoV-2–positive patients from Northern Italy. Physicians administered a survey-based questionnaire at recruitment (T0). During follow-up, patients responded to online dedicated surveys modulated according to symptoms at T0.

**Results:**

A total of 151 patients completed the follow-up survey. OD and/or GD were observed in 83% and 89% of subjects, respectively. Resolution rates of OD and GD at 30 days from onset were 87% and 82%, respectively. Risk factors for late resolution were grade of dysfunction at onset (total vs partial), gender, and presence of nasal congestion. Three (2%) patients previously reporting complete resolution of symptoms complained of subsequent recurrence of OD and/or GD after a mean of 19 days from resolution of the previous episode.

**Conclusion:**

COVID-19–related OD and GD had high rate of resolution in the first month from onset of symptoms. However, in 10% to 15% of patients, these symptoms showed only partial improvement after this period.

Recent reports have demonstrated a high prevalence of olfactory dysfunction (OD) and gustatory dysfunction (GD) in patients affected by COVID-19.^1-3^ Interestingly, both OD and GD appear to be weakly correlated with nasal congestion, which is suggestive of a primary chemosensory dysfunction conceivably due to SARS-CoV-2 neurotropism.^4-6^ Moreover, OD and GD in COVID-19 typically show sudden and early onset,^1-3,7^ with a higher prevalence in young, mildly symptomatic, female, nonsmoker, and healthy subjects.^3^ While the presence of these symptoms in COVID-19 has been widely demonstrated, data reported on their duration and resolution are still limited because the first epidemic peak has been only a few weeks ago in most Western countries.

In view of the large number of people affected worldwide, the definition of timing and persistence rate of OD and GD are a significant issue to be addressed because of their impact on quality of life.^8,9^

Contextually, we prospectively analyzed the clinical course of OD and GD in a cohort of home-quarantined patients who were previously recruited in a cross-sectional study.^3^ The aim of the study was to provide information on the changes in OD and GD in COVID-19, focusing on the rate and timing of resolution, and to correlate different patterns of resolution to specific clinical variables.

## Materials and Methods

A cohort of subjects under home quarantine for COVID-19 was recruited in an Italian region (Lombardy) with a high prevalence for SARS-CoV-2. All subjects were previously included in a cross-sectional study^3^ and followed-up by assessing the evolution of OD and GD. This report followed the Strengthening the Reporting of Observational Studies in Epidemiology (STROBE) guidelines for cohort studies.^10^ All subjects signed an informed consent form approved by the institutional review board. The study was performed following the principles of the Declaration of Helsinki and was approved by the Research Review Board, Ethics Committee of the ASST Spedali Civili of Brescia, Italy (study reference number: NP4037).

### Study Cohort, Setting, and Data Collection

Subjects home quarantined for SARS-CoV-2 infection demonstrated by nasal-pharyngeal swab were recruited through exponential snowball sampling from March 27 to April 1, 2020: each enlisted individual was asked to provide contact with new positive candidates, who were in turn recruited after giving their consent.

Study inclusion criteria were as follows:

- signed written informed consent,
- male or female >18 years of age,
- willing and able to participate in the study,
- positive nasal-pharyngeal swab for SARS-CoV-2 (reverse transcriptase polymerase chain reaction), and
- overall clinical status not requiring hospitalization.

Exclusion criteria were

- legal incapacity or limited legal capacity.
- medical or psychological condition or situation which in the opinion of the investigator would not permit the patient to complete the questionnaire or sign informed consent, and
- preexisting chronic anosmia and/or ageusia.

All subjects recruited were symptomatic because the national health care system policy indicated nasal-pharyngeal swab for SARS-CoV-2 only for individuals with referring symptoms that were suspicious for COVID-19. The quarantine was concluded after 2 negative nasal-pharyngeal swabs.

### Study Objectives

The primary objective was to determine the evolution of SARS-CoV-2–related OD and GD over time, with a particular focus on its rate of resolution at different time points. The secondary objective was to identify clinical characteristics associated with different timings of resolution of OD and GD.

### Survey-Based Questionnaires and Follow-up

At the time of recruitment (T0), a survey-based questionnaire (see the Supplemental Materials) on patient characteristics and symptoms with a focus on OD and GD (ie, type, intensity, and timing) was administered by a physician.^3^ Patients were followed by dedicated online surveys from April 27 to May 5, 2020 (Supplemental Materials). Each evaluation was modulated according to the state of OD and GD at T0, as follows:

- patients without OD or GD at T0 took a survey on the possible appearance of symptoms during follow-up,
- patients with ongoing OD or GD at T0 received a survey that focused on improvement or resolution of these symptoms (timing and degree of improvement), and
- patients in which OD or GD was resolved at T0 received a survey on the possible reemergence of symptoms during follow-up.

The degree of OD and GD was defined as “partial” or “total” to avoid subjective interpretations.

### Statistical Analysis

The analysis was limited to subjects reporting OD and/or GD. Time to recovery for OD and GD was estimated by Kaplan-Meier curves considering complete recovery as an endpoint. Differences among groups were evaluated using log-rank test and Cox proportional-hazard models as appropriate. Variables analyzed were age, gender, smoking history, comorbidities, other symptoms, grade of OD/GD (ie, partial vs total), and timing of presentation of OD and GD. Multivariable analysis was performed with Cox proportional hazards models including all variables with a *P* value <0.1 at univariate analysis.

## Results

A total of 213 patients were recruited. Of these, 151 (71%) completed both the initial survey-based questionnaire at T0 and subsequent follow-up form and were included in the final prospective cohort. The mean lag time between the first symptom onset and T0 survey was 22 days (SD, 7.6; range, 6-45 days). Patient characteristics are summarized in **Table 1**. No regional treatment protocol for COVID-19 and OD or GD in mildly symptomatic nonhospitalized subjects was available at the time of evaluation.

**Table 1.** Patient Characteristics.

	n (%)
Mean age, y (range)	45 (18-70)
Gender, n (%)	
Male	56 (37)
Female	95 (63)
Main comorbidities, n (%)	
Obesity	4 (3)
Hypertension	20 (13)
Asthma or allergic rhinitis	19 (13)
Cardiopathy	3 (2)
Diabetes	3 (2)
Immune disorders	3 (2)
Pneumopathy	2 (1)
Nephropathy	0 (0)
Number of comorbidities, n (%)	
0	105 (70)
1	30 (20)
2	11 (7)
3	5 (3)
Smoking history, n (%)	
Current smoker	12 (8)
Former smoker	26 (17)
Nonsmoker	113 (75)

OD and/or GD were observed in 126 (83%) and 135 (89%) subjects, respectively. OD and GD developed as the first symptom in 14% and 16% of cases, respectively. In the remaining cases, the mean time of onset after the first symptom was 3 days (SD, 2.7; range, 0-10 days) for OD and 3.2 days (SD, 2.7; range, 0-10 days) for GD.

### Olfactory Dysfunction

**Figure 1.**
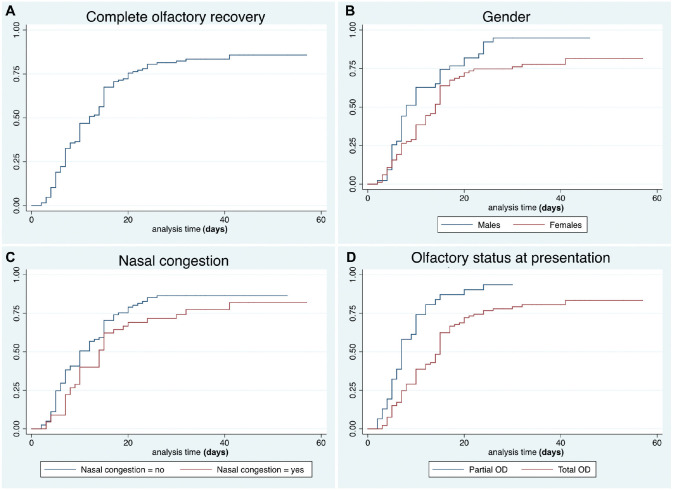
Kaplan-Meier curves showing the recovery pattern of olfactory dysfunction in the entire series (A), according to gender (B), nasal congestion (C), and grade of olfactory dysfunction at presentation (D).

Thirty-three (26%) patients reported partial OD and 93 (74%) anosmia. Complete recovery rates are reported in **Table 2** and **Figure 1**. A total of 20 (16%) subjects reported ongoing OD at the end of the follow-up (mean time from onset, 37 ± 9 days), of which 16 (80%) reported partial improvement.

**Table 2.** Recovery Rate of Olfactory Dysfunction at Different Time Points.

Follow-up (days from symptom onset)	Complete recovery rate (95% CI)
5	19.0% (27.1-13.2)
10	46.8% (55.9-38.5)
15	67.5% (75.5-59.3)
20	75.5% (82.6-67.7)
25	80.5% (87.0-73.2)
30	82.3% (88.5-75.1)
35	83.4% (89.5-76.2)
40	83.4% (89.5-76.2)

Late complete recovery was associated with total OD at presentation (*P* < .001) and female gender (*P* = .02). Association with nasal congestion was not statistically significant at univariate analysis (*P* = .1). At multivariable analysis, only total OD at presentation and nasal congestion were confirmed as independent variables (**Table 3**).

**Table 3.** Multivariable Analysis of Factors Associated With Timing of Recovery of Olfactory and Gustatory Dysfunction.^a^

Variable	Hazard ratio	*P* value	95% CI
Olfactory dysfunction			
Gender	0.74	.147	0.49-1.12
Grade of dysfunction	0.43	<.001	0.27-0.68
Nasal congestion	0.59	.016	0.38-0.90
Gustatory dysfunction			
Gender	0.66	.040	0.44-0.98
Grade of dysfunction	0.68	.083	0.44-1.05
Nasal congestion	0.73	.131	0.48-1.10

a Gender: male versus female; grade of dysfunction: partial versus total; nasal congestion: no versus yes.

### Gustatory Dysfunction

**Figure 2.**
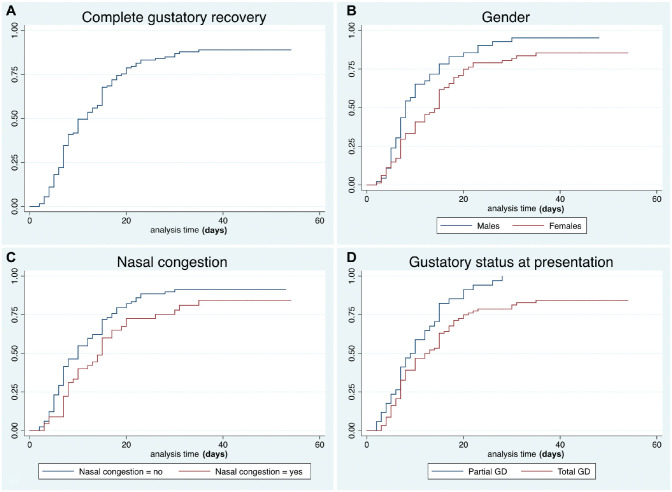
Kaplan-Meier curves showing the recovery pattern of gustatory dysfunction in the entire series (A), according to gender (B), nasal congestion (C), and grade of gustatory dysfunction at presentation (D).

Forty (30%) patients reported partial GD and 95 (70%) ageusia. Complete recovery rates are reported in **Table 4** and **Figure 2**. A total of 16 (12%) subjects reported ongoing GD at the end of the follow-up (mean time from onset, 33 ± 15 days), of which 11 (69%) reported partial improvement.

**Table 4.** Recovery Rate of Gustatory Dysfunction at Different Time Points.

Follow-up (days from symptom onset)	Complete recovery rate (95% CI)
5	18.1% (26.0-12.4)
10	49.6% (58.6-41.3)
15	67.7% (75.7-59.6)
20	78.7% (85.5-71.2)
25	83.2% (89.3-75.9)
30	86.8% (92.2-80.1)
35	88.9% (93.9-82.3)
40	88.9% (93.9-82.3)

Late complete recovery was associated with total GD at presentation (*P* = .006), female gender (*P* = .013), and presence of nasal congestion (*P* = .046). Only female gender was independently associated with late recovery at multivariable analysis (**Table 3**).

### Recurrence of Olfactory and Gustatory Dysfunction

Three (2%) patients previously reporting complete resolution of symptoms referred a subsequent recurrence of OD (1%; n = 2) and/or GD (1%; n = 2) at a mean of 19 ± 5 days after resolution of the previous episode. These alterations were still ongoing at the time of the evaluation without other symptoms related to COVID-19. All of these patients had 2 negative nasal-pharyngeal swabs before recurrence.

### Olfactory and Gustatory Dysfunctions and the Course of the Infection

One-hundred forty (93%) patients concluded the quarantine after 2 consecutive negative swabs. OD and GD recovered before the negative swabs in 101 (67%) and 98 (65%) cases, respectively, while they were still ongoing in 20 (13%) and 23 (15%) at the time of the second negative swab.

## Discussion

The present longitudinal study documents the mid-term evolution of OD and GD in a cohort of patients with confirmed COVID-19. The evaluation focused on the rate and timing of resolution of chemosensory dysfunction, specifically assessing factors associated with late recovery.

Some peculiar characteristics can be observed. The curves relative to our data clearly show a plateau pattern of recovery, with a flattening at approximately 80% after the 20th day from symptom onset. Similar results^7^ were reported in a recent paper assessing patients with OD and/or GD during the COVID-19 epidemic. Although the onset of OD and/or GD has been demonstrated to be almost concomitant with the first symptoms of the disease, their resolution is not strictly related to negativity of nasal-pharyngeal swabs. Therefore, OD and GD arguably may last beyond the resolution of the infection. In addition, we observed a recurrence of OD and GD after complete resolution of symptoms in 2% of patients. This is a novel observation that could shed some light on the etiopathogenetic mechanism of SARS-CoV-2 chemosensory dysfunction. Of note, all of these patients showed negative swabs before recurrence, but unfortunately, none repeated the swab after the relapse. However, the possibility that OD and/or GD may be directly correlated to viral load should not be overlooked and should be prospectively assessed by other series.

The grade of dysfunction (partial vs total) at onset was the most critical variable associated with the timing of resolution. In fact, patients presenting with a complete chemosensory dysfunction (anosmia or ageusia) had significantly longer recovery times.

Women showed slower recovery of both OD and GD. In GD, this was an independent factor that influenced the time of improvement. On one hand, this could simply reflect gender differences in completing voluntary questionnaires.^7^ On the other, in line with the higher incidence of OD and GD in women with COVID-19 reported by several authors,^1,3,4,11^ females may be characterized by an increased sensitivity to minor OD and GD,^8,9,12^ delaying the subjective threshold of “complete recovery.”

Although the loss of smell in COVID-19 has been defined as prevalently sensorineural, a transmissive component due to nasal congestion is independently associated with a delayed subjective recovery. However, its independent association with late improvement was not confirmed for GD, supporting the hypothesis that GD is not secondary to OD.

Interestingly, age was not associated with rates of recovery of chemosensory dysfunction. Nevertheless, the mean age of the series was particularly low (45 years) in accordance with the selection criteria of management by home quarantine. This aspect significantly reduces the impact of age-related hyposmia and hypogeusia.

Some limitations of the study should be underlined. While the response rate was greater than 70%, the influence of selection bias should not be overlooked. Symptomatic patients are significantly more likely to respond to follow-up surveys, and this could lead to an overestimation of disease prevalence. To partially address this issue, the analysis included only patients with chemosensory dysfunction, in line with the study objective (ie, evaluation of recovery). Furthermore, the study cohort was recruited at T0 by means of a cross-sectional survey. Therefore, symptom evaluation is partially retrospective (ie, before T0) and partially prospective (ie, after T0). For this reason, a recall bias could affect the precision of data collection. However, the first evaluation had been performed in the early epidemic phase, and the mean time from symptom onset to survey (T0) was limited. Although therapies that could potentially influence OD and GD were ruled out at T0, specific evaluation of empiric treatments for OD and GD was not performed during the follow-up. In fact, no treatment protocol was instituted because of the limited access to medical facilities for nonurgent complaints. Lastly, the entire analysis was based on subjective questionnaires. On this basis, further long-term prospective studies aimed at objectively analyzing and characterizing the evolution of OD and GD are warranted.

## Conclusions

COVID-19–related OD and GD had high rates of resolution in the first month after symptom onset. However, 10% to 15% of patients do not report complete resolution of these symptoms even after 45 days. The main risk factors for late resolutions are grade of dysfunction at onset (total vs partial), gender, and presence of nasal congestion. Objective tests may help to clarify these different patterns of resolution and to better characterize patients presenting a late recovery or persistent symptoms in the long term.

## Supplemental Material

Supplementary_Materials – Supplemental material for Olfactory and Gustatory Outcomes in COVID-19: A Prospective Evaluation in Nonhospitalized Subjects Click here for additional data file. Supplemental material, Supplementary_Materials for Olfactory and Gustatory Outcomes in COVID-19: A Prospective Evaluation in Nonhospitalized Subjects by Alberto Paderno, Davide Mattavelli, Vittorio Rampinelli, Alberto Grammatica, Elena Raffetti, Michele Tomasoni, Tommaso Gualtieri, Stefano Taboni, Silvia Zorzi, Francesca Del Bon, Davide Lombardi, Alberto Deganello, Luca Oscar Redaelli De Zinis and Alberto Schreiber in Otolaryngology–Head and Neck Surgery
