# Supplementary material for: Olfactory and Gustatory Outcomes in COVID-19: A Prospective Evaluation in Nonhospitalized Subjects
Source: Otolaryngol Head Neck Surg. 2020 Jun 30;163(6):1144–9. doi: 10.1177/0194599820939538 (PMC7331108; doi:10.1177/0194599820939538)
Supplement: Supplementary_Materials – Supplemental material for Olfactory and Gustatory Outcomes in COVID-19: A Prospective Evaluation in Nonhospitalized Subjects [file Supplementary_Materials.docx]

**Supplementary Materials**

These Supplementary Materials have been provided by the authors to give readers additional information about their manuscript.

Supplement to: Paderno A, Mattavelli D, Rampinelli V, et al. OLFACTORY AND GUSTATORY OUTCOMES IN COVID-19: A PROSPECTIVE EVALUATION.

**Table of contents**

| 1. | Methods. Survey-based questionnaire administered by physicians at T0…..………………………….. | pg.3 |
| --- | --- | --- |
| 2. | Methods. On-line questionnaires…..…………………………………………………………………… | pg.5 |

**1. Methods. Survey-based questionnaire administered by physicians at T0.**

The interview questionnaire was defined and administered in order to minimize specific biases (i.e., extreme responding, question/response order). Patients were not informed about the specific goal of the study to minimize confirmation bias. Herein, the translated versions of the questionnaire and data collection form are reported.

**Questions administered**

1. What symptoms do you have or have had? I will tell you a list of symptoms and you need to answer “yes” or “no”.

2. Do you have or have had other symptoms?

3. What was the first symptom you had?

4. How many days ago was the first symptom?

5. (If olfactory dysfunction present) When did problems with smell start?

6. (If gustatory dysfunction present) When did problems with taste start?

7. Was the loss of smell partial or total?

8. Was the loss of taste partial or total?

9. Has the loss of smell improved or resolved? After how long?

10. Has the loss of taste improved or resolved? After how long?

**Data collection form**

Patient status: □ Hospitalized □ Quarantined Date:………………………….

Name:……………….. Surname:…………………

Date of birth:…………………………………………………. Gender: □ M □ F

PATIENT HISTORY DATA COLLECTION

Smoker: □ YES cig/day……since….…..years □ NO □ FORMER SMOKER since………years cig/die….….. for…….years

Date of swab for SARS-Cov-2:…….

Comorbidities:

| □ Obesity | □ Hypertension | □ Cardiac disease | □ Diabetes mellitus |
| --- | --- | --- | --- |
| □ Renal disease | □ Chronic rhinosinusitis/asthma | □ Pulmonary disease | □ Other…………………….. |

Symptoms (also mark the first symptoms in the right box with an X):

| □ dysosmia (before oxygen therapy) |  | □ cough |  | □ asthenia |  | □ nausea (before medical treatment) |  | □ ocular discomfort (before oxygen therapy) |  |
| --- | --- | --- | --- | --- | --- | --- | --- | --- | --- |
| □ dysgeusia (before oxygen therapy) |  | □ dyspnoea |  | □ arthromyalgia |  | □ nasal congestion (before oxygen therapy) |  | □ syncope |  |
| □ fever |  | □ headache |  | □ diarrhoea (before medical treatment) |  | □ pharyngodynia (before oxygen therapy) |  | □ Other…………….. |  |

- Date of the first symptom(s): ……

- Grade of olfactory dysfunction: □ none □ partial □ total comments: ………………………………….

Days from the first symptom to the onset of olfactory dysfunction:….

If olfactory dysfunction was the first symptom, after how many days did the second symptom appear?

Duration of olfactory dysfunction (days): □ still present □ resolved

- Grade of gustatory dysfunction: □ none □ partial □ total comments: ………………………………….

Days from the first symptom to the onset of gustatory dysfunction:….

If gustatory dysfunction was the first symptom, after how many days did the second symptom appear?

Duration of gustatory dysfunction (days): □ still present □ resolved

**2. Methods. On-line questionnaires.**

The questionnaires were defined and administered in order to minimize specific biases (i.e., extreme responding, question/response order). Patients were not informed about the specific goal of the study to minimize confirmation bias. Herein, the translated versions of the questionnaire and data collection form are reported.

**Questions administered (possible answers)**

**Group 1.** Patients without olfactory and gustatory dysfunction at T0

1. Please complete the general information form

Name

Surname

Date of birth

1. Have you experienced any new dysfunction in your sense of smell?

Yes, complete loss

Yes, partial loss

No

1. If yes, did the new dysfunction of the smell resolve?

Yes, completely resolved

No, persistent

No, but improving

1. If yes to question 2, enter the start and end date of the new dysfunction in your sense of smell.

Date

1. Have you experienced any new dysfunction in your sense of taste?

Yes, complete loss

Yes, partial loss

No

1. If yes, did the new dysfunction of the sense of taste resolve?

Yes, completely resolved

No, persistent

No, but improving

1. If yes to question 5, enter the start and end date of the new dysfunction in your sense of taste.

Date

1. Please enter the date of the first positive swab and the quarantine end date (second negative swab).

Date first positive

Date second negative swab

**Group 2.** Patients with ongoing olfactory and gustatory dysfunction at T0

1. Please, complete the general information form

Name

Surname

Date of birth

1. Do you still have smell dysfunctions?

Yes, complete loss

Yes, partial loss

No, complete resolution

1. If completely resolved, when did you start to smell normally again?

Date

1. Was the resolution gradual (over several days) or sudden (over 1-2 days)?

Gradual

Sudden

1. Do you still have taste dysfunctions?

Yes, complete loss

Yes, partial loss

No, complete resolution

1. If completely solved, when did you start to taste normally again?

Date

1. Was the resolution gradual (over several days) or sudden (over 1-2 days)?

Gradual

Sudden

1. Are you still under quarantine (waiting for the second negative swab)?

Yes

No

1. If the quarantine is over, enter the quarantine end date (second negative swab).

Date

1. Do you want to report anything else (unusual olfactory and gustatory sensations)?

**Group 3.** Patients in which olfactory and gustatory dysfunction were resolved at T0

1. Please complete the general information form

Name

Surname

Date of birth

1. Have you experienced any new dysfunction in your sense of smell?

Yes, complete loss

Yes, partial loss

No

1. If yes, enter the start date.

Date

1. If yes to question 2, did the new dysfunction of the sense of smell resolved?

Yes, completely resolved

No, persistent

No, but improving

1. If completely or almost completely resolved, when did you start to smell normally again?

Date

1. Have you experienced any new dysfunction in your sense of taste?

Yes, complete loss

Yes, partial loss

No

1. If yes, enter the start date.

Date

1. If yes to question 6, did the new dysfunction of the sense of taste resolved?

Yes, completely resolved

No, persistent

No, but improving

1. If completely or almost completely resolved, when did you start to taste normally again?

Date

1. If the quarantine is over, enter the quarantine end date (second negative swab) - do not enter if still ongoing.

Date
